# Selenium level correlates negatively with antibodies but positively with thyroid function in children with down syndrome: an Indonesian study

**DOI:** 10.3389/fendo.2023.1177373

**Published:** 2023-05-10

**Authors:** Yuni Hisbiyah, Anang Endaryanto, Bagus Setyoboedi, Nur Rochmah, Muhammad Faizi, Katherine Fedora

**Affiliations:** ^1^ Doctoral Program of Medical Science, Faculty of Medicine, Universitas Airlangga, Surabaya, East Java, Indonesia; ^2^ Faculty of Medicine, Department of Child Health, Dr. Soetomo General Hospital, Universitas Airlangga, Surabaya, East Java, Indonesia; ^3^ Faculty of Medicine, Universitas Airlangga, Surabaya, East Java, Indonesia

**Keywords:** selenium, autoimmune, thyroid, down syndrome, Indonesia

## Abstract

**Background:**

Children with Down syndrome (DS) are prone to developing autoimmune thyroid disease (AITD). Previous studies found lower selenium (Se) levels in children with AITD. Glutathione peroxidase-3 (GPx3) and selenoprotein-P (SePP) are widely used to measure Se levels. DS children tend to have lower Se levels, the main contributor to hypothyroidism in this population. This study aimed to analyze the Se’s role in AITD in Indonesian children with DS.

**Methods:**

This cross-sectional study was conducted between February 2021-June 2022 at the Pediatric Outpatient Clinic of Dr Soetomo Hospital. DS children aged 1 month to 18 years were enrolled using consecutive sampling. Thyroid-stimulating hormone, free thyroxine, thyroid peroxidase (TPO-Ab) and thyroglobulin (Tg-Ab) autoantibody, GPx3, and SePP levels were measured in plasma samples using enzyme-linked immunosorbent assays. Statistical analyses used Chi-square, Mann–Whitney, and Spearman’s rank correlation (*r*_s_). All results with *p*<0.05 were considered statistically significant.

**Results:**

Among 62 children with DS, SePP and GPx3 levels were significantly lower in those with AITD than those without AITD (*p*=0.013 and *p*=0.018, respectively). SePP and GPx3 levels correlated significantly with lower TPO-Ab (*r*_s_=−0.439 with *p*=1×10^-5^ and *r*_s_=−0.396 with *p*=0.001, respectively) and Tg-Ab (*r*_s_=−0.474 with *p*=1×10^-5^ and *r*_s_=−0.410 with *p*=0.001, respectively) levels. SePP levels correlated significantly with lower thyroid dysfunction incidence (*r*_s_=-0.252, *p*=0.048) in the AITD group.

**Conclusion:**

Selenium deficiency contributes to autoimmune process in the thyroid and to thyroid dysfunction in children with Down syndrome. Our findings recommend increasing Se levels through Se-containing foods to reduce the risks of AITD and thyroid dysfunction in DS children with AITD.

## 1 Introduction

Autoimmune thyroid disease (AITD) is the most frequent cause of acquired thyroid dysfunction, presenting either as Hashimoto’s thyroiditis (HT) or Graves’ disease (GD). AITD arises from the complex interplay between genetic and environmental such as micronutrient deficiency, leading to loss of self-tolerance to thyroid antigens and production of antibodies such as thyroid peroxidase (TPO) and thyroglobulin (Tg) (1).

Selenium (Se) is a trace element that is known to play a role in the thyroid metabolism. The thyroid gland has higher Se concentrations than other organs (2). Se’s biological effects are mediated by Se-containing proteins (SePs) (3–5) which are widely measured in the form of selenoprotein-P (SePP) and gluthation peroxidase-3 (GPx3) (6–8). SePP functions in Se transport and antioxidant defence, while GPx is an antioxidant enzyme that removes hydrogen peroxide (H_2_O_2_) and protects thyroid cells from oxidative damage (9, 10). Se deficiency may increase autoimmunity risk (11). Several studies reported lower selenium (Se) levels in children with AITD (12–14).

Patients with Down syndrome (DS) are prone to developing autoimmune disorders, with the most frequent is autoimmune hypothyroidism. DS children experience increased oxidative stress due to the overexpression of several chromosome 21 genes, including copper-zinc superoxide dismutase 1 (*SOD1*) (15). Therefore, DS patients may have below-normal plasma Se levels (16, 17) due to the high demand for Se in SeP (GPx) production induced by high SOD-mediated H_2_O_2_ production (17). Indonesian children have low Se levels (18, 19). In addition, Se content of rice, a staple food in Indonesia, is lower than in other countries (20). While many studies have shown an association between Se deficiency and AITD in adults and children in the general population, until recently, no published studies have examined in DS population. Therefore, this study examines Se’s role in thyroid autoimmunity and function in Indonesian children with DS.

## 2 Materials and methods

### 2.1 Study participants

This cross-sectional study was conducted at the Pediatric Outpatient Clinic of Child Health at the Dr Soetomo General Hospital in Surabaya, Indonesia, between February 2021 and June 2022. This study was approved by the Institutional Review Board of Dr Soetomo General Hospital (0397/KEPK/III/2022). The parents of participants provided informed consent. It included 62 DS children aged between one month and 18 years enrolled using consecutive sampling. The inclusion criteria were patients regularly visiting the hospital during the study and parental informed consent to participate. The exclusion criteria were mother with AITD, children with acute medical conditions, severe malnutrition, or history of micronutrient deficiencies.

This study used a self-reported questionnaire completed by parents to record participants’ sex, age, and sociodemographic status. DS was diagnosed through karyotyping. Thyroid function was classified as hypothyroidism, hyperthyroidism, or euthyroidism based on typical free thyroxine (FT4) and thyroid-stimulating hormone (TSH) values (21). Positive autoimmune thyroid antibodies against TPO (TPO-Ab) or Tg (Tg-Ab) with or without thyroid function problems formed the basis for AITD diagnosis (22). Se levels were assessed based on SePP and GPx3 levels. TSH, FT4, TPO-Ab, Tg-Ab, SePP, and GPx3 levels were measured in plasma samples.

### 2.2 Plasma measurements

#### 2.2.1 TPO-Ab and Tg-Ab levels

TPO-Ab and Tg-Ab levels were measured using commercial enzyme-linked immunosorbent assays (ELISA) obtained from Demeditec Diagnostics GmbH: TPO-Ab ELISA kit (Cat. No. DE7580) and Tg-Ab ELISA kit (Cat. No. DE7590). The manufacturer-specified threshold values were used to interpret the autoimmune thyroid markers: >75 IU/mL (positive), 50–75 IU/mL (borderline), and <50 IU/mL (negative) for TPO-Ab, and >150 IU/mL (positive), 100-150 IU/mL (intermediate), and 100 IU/mL (negative) for Tg-Ab.

#### 2.2.2 FT4 and TSH levels

Thyroid function was assessed based on FT4 (Cat. No. CAN-FT4-4340) and TSH (Cat. No. CAN-TSH-4080) levels measured using commercial ELISA kits obtained from DBC-Diagnostics Biochem Canada Inc. The reference values were those reported by Sperling et al. for pediatric care (23).

#### 2.2.3 Se levels

Se levels were measured using commercial ELISA kits for human SePP (Cat. No. E2196Hu) and GPx3 (Cat. No. E3922Hu) obtained from BT Lab.

### 2.3 Statistical analyses

Kolmogorov–Smirnov and Levene tests were used to assess the normality and homogeneity of the data, respectively. Participants were placed into two groups: AITD and non-AITD. Descriptive analyses compared the demographic characteristics of DS patients in the AITD and non-AITD groups. SePP, GPx3, TPO-Ab, and Tg-Ab levels were compared between the AITD and non-AITD groups using Chi-square, and Mann–Whitney tests. Spearman’s rank correlation coefficient (*r*_s_) was used to assess the correlation between Se levels and AITD status. All results with *p*<0.05 were considered statistically significant.

## 3 Results

This study involved 62 DS patients, of which 48 had positive thyroid autoimmune markers (TPO-Ab and/or Tg-Ab) and were placed in the AITD group, while the remaining 14 were placed in the non-AITD group. Patients in the AITD group were younger at enrolment than those in the non-AITD group. Standardised body mass index (BMI-SDS) did not differ significantly between groups. Congenital abnormalities and thyroid dysfunction were more common in the AITD group than in the non-AITD group. The demographic characteristics of the subjects in each group are shown in **Table 1**.

**Table 1:** Demographic characteristics of each group.

Characteristic	AITD (*n* = 48)	Non-AITD (*n* = 14)	*p*
**Age at enrollment (months; median [min–max])**	18.5 (1–175)	20.5 (1–130)	0.860^M^
**BMI-SDS (mean ± SD)**	14.827 ± 2.954	16.551 ± 3.519	0.750^M^
Sex (n, %)			
**Male**	28 (80.0%)	7 (20.0%)	0.054^C^
**Female**	19 (73.0%)	7 (26.9%)	
Prematurity history (n, %)			
**No**	43 (78.2%)	12 (21.8%)	0.164^C^
**Yes**	4 (66.7%)	2 (33.3%)	
Mother’s education level (n, %)			
**Elementary school**	2 (66.7%)	1 (33.3%)	0.705^C^
**Junior high school**	4 (100%)	0 (0%)	
**Senior high school**	26 (76.5%)	8 (23.5%)	
**Bachelor (higher education)**	16 (76.2%)	5 (23.8%)	
Associated congenital malformation (n, %)			
**Yes**	28 (93.3%)	2 (6.7%)	0.004^C*^
**No**	20 (62.5%)	12 (37.5%)	
Thyroid function (n, %)			
**Hypothyroid**	32 (88.9%)	4 (11.1%)	0.003^C*^
**Hyperthyroid**	5 (100%)	0 (0%)	
**Euthyroid**	11 (52.4%)	10 (47.6%)	
LT4 therapy			
**Yes**	27 (56.25%)	4 (28.6%)	0.068^F^
**No**	21 (43.75%)	10 (71.4%)	
**LT4 therapy duration (months; median [min–max])**	12 (1–96)	11.5 (5–24)	0.929^M#^

^C^, Chi-square test; ^F^, Fisher’s exact test; ^M^, Mann–Whitney test; *p<0.05; ^#^, statistical analysis performed using the 31 participants receiving LT4 therapy (27 in the AITD and four in the non-AITD group).

Bold values are the results of p-value calculations which show significant results with p <0.05.

Mean TPO-Ab and Tg-Ab levels were significantly higher in the AITD group than in the non-AITD group. Median SePP and GPx3 levels were significantly lower in the AITD group than in the non-AITD group. The laboratory descriptions of Se (SePP and GPx3) and thyroid autoantibody (TPO-Ab and Tg-Ab) levels are presented in **Table 2**.

**Table 2:** Se, TPO-Ab, and Tg-Ab levels in each group.

Characteristics	AITD (*n* = 48)	Non-AITD (*n* = 14)	*p*
**Se level (ng/mL; median (min–max))** **SePP** **GPx3**	24.81 (6.72–228.76) 1.39 (0.12–36.97)	91.68 (11.01–186.51) 7.62 (0.78–20.69)	0.013^M*^ 0.018^M*^
**TPO-Ab level** **(IU/mL; median (min–max))**	262.62 (6.64–7989.14)	22.38 (5.02–29.80)	1×10^-5 M*^
**Tg-Ab level** **(IU/mL; median (min–max))**	938.00 (52.40–9746.00)	22.55 (6.10–110.30)	1×10^-5 M*^

^M^, Mann–Whitney test; *p<0.05.

Bold values are the results of p-value calculations which show significant results with p <0.05.

SePP and GPx3 levels were higher in AITD patients with normal thyroid function than with thyroid dysfunction. However, only the SePP difference was significant (*p*=0.049). More detailed information on the correlations between Se levels and thyroid function in the two groups is provided in **Table 3**.

**Table 3:** Relationship between Se levels and thyroid function in DS children with AITD.

Variable	Thyroid dysfunction (n = 38)		Euthyroid (n = 11)	*p*
	Hypothyroidism (n = 32)	Hyperthyroidism (n = 5)		
**SePP level** **(ng/mL; median [min–max])**	24.814 (6.720–221.660)	30.955 (14.270–58.010)	69.473 (8.160–228.76)	0.049 ^M*^
**GPx3 level** **(ng/mL; median [min–max])**	1.465 (0.241–34.639)	1.374 (0.543–2.398)	5.771 (0.115–36.969)	0.252 ^M^

^M^, Mann–Whitney test; *p<0.05.

Bold values are the results of p-value calculations which show significant results with p <0.05.

SePP levels were significantly negatively correlated with AITD incidence (*r*_s_=−0.319, *p*=0.011) and TPO-Ab (*r*_s_=−0.439, *p*=1×10^-5^) and Tg-Ab (*r*_s_=−0.396, *p*=0.001) levels. GPx3 levels were also significantly negatively correlated with AITD incidence (*r*_s_=−0.302, *p*=0.017) and TPO-Ab (*r*_s_=−0.474, *p*=1×10^-5^) and Tg-Ab (*r*_s_=−0.410, *p*=0.001) levels. The correlations between SePP and GPx3 levels and AITD status and mean thyroid antibody levels are shown in **Table 4**.

**Table 4:** Correlations between Se levels and AITD status and TPO-Ab and Tg-Ab levels.

Variable	AITD status		TPO-Ab level		Tg-Ab level	
	*p*	*r*_s_	*p*	*r*_s_	*p*	*r*_s_
**SePP level**	0.011^*^	−0.319	1×10^-5 *^	−0.439	0.001^*^	−0.396
**GPx3 level**	0.017^*^	−0.302	1×10^-5 *^	−0.474	0.001^*^	−0.410

*p<0.05.

Bold values are the results of p-value calculations which show significant results with p <0.05.

The correlations between SePP and GPx3 levels and thyroid function are shown in **Table 5**. While SePP levels were significantly negatively correlated with thyroid dysfunction incidence in AITD children, GPx3 levels were not significantly correlated.

**Table 5:** Correlations between Se levels and thyroid function in the AITD group.

Variable	Thyroid function	
	*p*	*r*_s_
**SePP level**	0.048*	−0.252
**GPx3 level**	0.255	−0.147

*p<0.05.

Bold values are the results of p-value calculations which show significant results with p <0.05.

## 4 Discussion

Our study showed that DS children with AITD had considerably lower Se levels than children without AITD based on SePP and GPx3 levels. We cannot compare these findings with other studies since none have examined Se levels and AITD in children with DS. Two population-based cross-sectional Chinese studies reported that AITD prevalence was significantly lower in adequate-Se than in the low-Se areas (24, 25). Another Danish population study also reported similar results (26). Several studies reported lower Se levels in AITD patients than in controls (27–29). While studies in children remain very limited, several have reported that children with HT have lower Se levels than controls (30, 31). As the main SeP involved in circulating Se transport (>50% of blood-borne Se), SePP levels strongly reflect circulating Se levels (7), while GPx3 is the only extracellular GPx family member and underlies all the GPx activity in the plasma, protecting cells against reactive oxygen species in the extracellular environment. A study in adults newly diagnosed with AITD (GD and HT) showed that GPx3 activity was significantly lower in GD and HT patients than in controls (*p*<0.01 and *p*<0.001, respectively) (32). In contrast, Rostami et al. reported higher GPx3 activity in adults newly diagnosed with HT than in controls and in Se-deficient patients compared to Se-sufficient patients (29).

In this study, SePP and GPx3 levels were significantly negatively correlated with TPO-Ab and Tg-Ab levels and AITD incidence. One study on adults reported that serum Se levels were negatively correlated with serum TPO-Ab (*r*=−0.161, *p*=0.021) and Tg-Ab (*r*=−0.237, *p*=0.001) levels (24). Another adult study showed that higher serum Se levels were associated with significantly lower odds ratios (ORs) for AITD (0.47; 95% confidence interval: 0.35–0.65) (24). Mseddi et al. reported negative correlations between Se and Tg-Ab levels and TR-Ab levels in GD patients (r=−0.71 with *p*<0.05 and *r*=−0.73 with *p*<0.05, respectively) (32). They also reported a positive correlation between Se levels and GPx activity in HT patients (*r*=0.64, *p*<0.01), hypothesising that Se deficiency may be one main cause of low GPx activity in HT patients (32). The selenoenzyme GPx protects the thyroid from H2O2, a crucial co-factor for TPO’s catalysis of the iodination and coupling of tyrosyl residues in Tg to produce thyroid hormones (32). Decreased glutathione levels appear to be a specific parameter related to oxidative stress activation and development in HT since oxidative stress is associated with thyroid hormone deficiency, inflammation, and autoimmune parameters (33).

Our study showed that adequate SePP levels were significantly correlated with decreased thyroid dysfunction incidence in AITD. Limited studies on children reported that Se levels were found to be within normal limits in those with AITD and normal thyroid function (34, 35). However, another study found that GPx activity and SePP levels were non-significantly lower in AITD patients with hypothyroidism undergoing LT4 therapy (30). Our findings could be due to the fact that several SePs are required for thyroid hormone synthesis, so that Se deficiency directly impairs thyroid function (36), as demonstrated by a population study in China showed that populations with normal Se levels had lower prevalences of subclinical hypothyroidism, hypothyroidism and thyroid enlargement compared to the population with low Se levels (4).

In addition to these direct thyroid synthesis mechanisms, adequate Se levels in patients with AITD can modify the inflammatory and immune responses (37, 38) and are thought to be associated with improvements in thyroid function. A 6-month study of Se supplementation showed that Se levels significantly correlated with GPx3 (*r*=0.325, *p*=0.002), SePP (*r*=0.225, *p*=0.033) levels and changes in TPO-Ab (*r*=−0.278, *p*=0.008), Tg-Ab (*r*=−0. 437, *p*=0.003), TSH (*r*=−0.314, *p*<0.001), and Treg (*r*=0.275, *p*=0.009) levels (39). Se supplementation at a dose of 100 g/day for 6 months was reported to significantly reduce TPO-Ab levels and improve thyroid function in a meta-analysis of children with AITD (40). Se supplementation for 6 months in adult with HT showed beneficial effect on thyroid autoantibodies and thyroid function by increasing the antioxidant activity and upregulating the activated Treg cells. In addition, in subgroup analysis, subclinical HT that not receive LT4 therapy may benefit more from this treatment in the decrease of TSH levels (41). Selenium intake was aimed at achieving maximal GPx activity in plasma or erythrocytes (42), however it is suspected that GPx activity in humans is not sensitive to changes in Se supply unless the person has low baseline Se levels (43).

In this study, we found no significant correlation between GPx3 and thyroid dysfunction in our AITD patients. Several research groups have found discrepant GPx3 activity in HT patients compared to controls, possibly due to the variability and complexity of mechanisms regulating oxidative stress in humans (28). The substrate being used to measure GPx activity was also reported to have a role in the difference results. GPx measurement in red blood cells being more sensitive than plasma (44, 45).

In this study, the median age of DS children suffering from AITD was 18.5 (1-175) months. In between them, 17 children were less than one year old. Children with DS are more likely to have AITD at a younger age than the population average (46). Several studies reported that AITD is rare in children under three years (47, 48) and commonly diagnosed at eight years (49). However, one case series reported that thyroid antibodies had been detected in DS infants aged five months and eight months (50). In addition, a study by Johnson et al., 2019 reported that trisomy 21 was the cause of permanent neonatal diabetes, with median onset at diabetes diagnosis was 2.3 (0.4, 7.5) weeks, and 44% of them had positive islet autoantibodies at 4 month-10 years old (51). Those studies supported data that autoimmunity can already occur in infants with Down syndrome as the most common genetic syndrome associated with immune dysregulation, involving both innate and adaptive immunity.

A major strength of this study is that it is the first to examine Se status in DS children with AITD. Our findings show that DS children with AITD had considerably lower Se levels than children without AITD based on SePP and GPx3 levels. SePP levels not only correlated with lower antibody levels but also with thyroid dysfunction, thus it is necessary to consider a policy to optimise Se levels in AITD patients with thyroid dysfunction in addition to LT4 therapy. These results can be used as a reference for further studies with larger sample sizes to achieve statistical significance. It can also be used as a basis for formulating recommendations to maintain adequate Se levels in DS Children with AITD in Indonesia, a country with staple foods low in Se. The weaknesses of this study were that it was cross-sectional and could not assess causality. This study was limited to a single centre, and its results may not generalise to the entire population. Therefore, further studies with larger sample sizes and different study designs are needed to provide consistent data on the role of Se on thyroid autoimmunity in DS children with AITD.

## 5 Conclusion

This study showed that Se, through its function in antioxidant defense, played an essential role in reducing AITD. Monitoring Se levels and promoting a diet high in Se should be areas of focus to optimize outcomes in children with DS who are physiologically susceptible to lower Se levels and a higher risk of developing AITD than children without DS. This research can also be used to formulate recommendations for maintaining adequate Se levels in DS children with AITD in Indonesia, a country with low-Se staple foods. To provide consistent data on the role of Se on thyroid autoimmunity in DS children with AITD, additional studies with larger sample sizes and different study designs are required.

## Data availability statement

The datasets presented in this article are not readily available. Requests to access the datasets should be directed to Anang Endaryanto anang.endaryanto@fk.unair.ac.id.

## Ethics statement

The studies involving human participants were reviewed and approved by Institutional Review Board of Dr Soetomo General Hospital (0397/KEPK/III/2022). Written informed consent to participate in this study was provided by the participants’ legal guardian/next of kin.

## Author contributions

YH is responsible for the study’s conception, design, report drafting, and supervision. NR is responsible for the study’s design, drafting, data collection, and analysis of the data. MF has supervised the realization, evaluation of ethical aspects of regulation, and literature analysis. KF contributed to conducting the study, analysing the literature and data. AE contributed to the approval of the final draft, the analysis of data, and the literature. BS contributed to drafting and revising the article and also proofread the manuscript. All authors contributed to the article and approved the submitted version.

## References

[1] Kyritsi EM Kanaka-Gantenbein C. Autoimmune thyroid disease in specific genetic syndromes in childhood and adolescence. Front Endocrinol (Lausanne) (2020) 19:543(11). doi: 10.3389/fendo.2020.00543 PMC7466763 32973676

[2] Köhrle J Jakob F Contempré B Dumont JE. Selenium, the thyroid, and the endocrine system’. Endocr Rev (2005) 26(7):944–84. doi: 10.1210/er.2001-0034 16174820

[3] Duntas LH. Selenium and the thyroid: a close-knit connection. J Clin Endocrinol Metab (2005) 95(12):5180–8. doi: 10.1210/jc.2010-0191 20810577

[4] Rayman MP. Selenium and human health. Lancet (2012) 379(9822):1256–68. doi: 10.1016/S0140-6736(11)61452-9 22381456

[5] Duntas LH Benvenga S. Selenium: an element for life. Endocrine (2015) 48(3):756–75. doi: 10.1007/s12020-014-0477-6 25519493

[6] Duffield AJ Thomson CD Hill KE Williams S. An estimation of selenium requirements for new Zealanders. Am J Clin Nutr (1999) 70(5):896–903. doi: 10.1093/ajcn/70.5.896 10539752

[7] Hurst R Armah CN Dainty JR Hart DJ Teucher B Goldson AJ. Establishing optimal selenium status: results of a randomized. Am J Clin Nutr (2010) 91(4):923–31. doi: 10.3945/ajcn.2009.28169 PMC2844680 20181815

[8] Xia Y Hill KE Byrne DW Xu J Burk RF. Effectiveness of selenium supplements in a low-selenium area of China. Am J Clin Nutr (2005) 81(4):829–34. doi: 10.1093/ajcn/81.4.829 15817859

[9] Reddy VS Gouroju S Suchitra MM Suresh V Sachan A Srinivasa Rao PVLN. Antioxidant defense in overt and subclinical hypothyroidism. Horm Metab Res (2013) 45(10):754–8. doi: 10.1055/s-0033-1348262 23828125

[10] Schmutzler C Mentrup B Schomburg L Hoang-Vu C Herzog V Köhrle J. Selenoproteins of the thyroid gland: expression, localization and possible function of glutathione peroxidase 3. Biol Chem (2007) 388(10):1053–9. doi: 10.1515/BC.2007.122 17937619

[11] Barbati ZR. The role of the selenoprotein glutathione peroxidase-1 in T cell activation and differentiation. United States: Faculty of the Harvard Medical School, Harvard University (2016).

[12] Evliyaoğlu O Acar M Özcabı B Erginöz E Bucak F Ercan O. Vitamin d defciency and hashimoto’s thyroiditis in children and adolescents: a critical vitamin d level for this association? JCRPE (2015) 7(2):128–33. doi: 10.4274/jcrpe.2011 PMC4563184 26316435

[13] Wu Q Rayman MP Lv H Schomburg L Cui B Gao C. Low population selenium status is associated with increased prevalence of thyroid disease. J Clin Endocrinol Metab (2015) 100(11):4037–47. doi: 10.1210/jc.2015-2222 26305620

[14] Sönmezgöz E Ozer S Yilmaz R Önder Y Bütün I Bilge S. Hypovitaminosis d in children with hashimoto’s thyroiditis. Rev Med Chil (2016) 144(5):611–6. doi: 10.4067/s0034-98872016000500009 27552012

[15] Benzi G Moretti A. Age and peroxidative stress-related modifications of the cerebral enzymatic activities linked to mitochondria and the glutathione system. Free Radical Biol Med (1995) 19(1):77–101. doi: 10.1016/0891-5849(94)00244-E 7635361

[16] Kadrabová J Madáric A Sustrová M Ginter E. Changed serum trace element profile in down’s syndrome. Biol Trace Elem Res (1996) 54(3):201–6. doi: 10.1007/bf02784431 8909693

[17] Kanavin ØJ Aaseth J Birketvedt GS. Thyroid hypofunction in down’s syndrome: is it related to oxidative stress? Biol Trace Elem Res (2000) 78(1–3):35–42. doi: 10.1385/BTER:78:1-3:35 11314986

[18] Ihsan N Nurcahyani YD. Hubungan defisiensi selenium dengan thyroid stimulating hormone (TSH), triiodothyronin (T3), dan free thyroxine (Ft4) pada anak sekolah dasar di daerah endemik GAKI. Media Gizi Mikro Indonesia (2015) 6(2):123–32. doi: 10.22435/mgmi.v6i2.4519.123-132

[19] Mahayati W. Defisiensi selenium (Se) sebagai faktor risiko dilated cardiomyopathy (DCM) di rumah sakit umum pusat (RSUP) sanglah, Bali: studi kasus – kontrol. e-Jurnal Medika (2019) 8(5):1–7.

[20] Holik HA Bianti H Mutakin M Abdulah R. Determination of selenium concentration in different species of rice consumed in bandung Indonesia. Int Res J Pharm App Sci (2013) 3(3):38–41. doi: 10.25220/WNJ.V03.i2.0004

[21] Cooper DS Biondi B. Subclinical thyroid disease. Lancet [Internet] (9821) 2012:1142–54:379. doi: 10.1016/S0140-6736(11)60276-6 22273398

[22] Nicholson LB Wong FS Ewins DL Butler J Holland A Demaine AG. Susceptibility to autoimmune thyroiditis in down’s syndrome is associated with the major histocompatibility class II DQA 0301 allele. Clin Endocrinol (1994) 41(3):381–3. doi: 10.1111/j.1365-2265.1994.tb02561.x 7955446

[23] Sperling MA. Pediatric endocrinology, 5th Edition. United States: Elsevier (2020).

[24] Teng X Shan Z Chen Y Lai Y Yu J Shan L. More than adequate iodine intake may increase subclinical hypothyroidism and autoimmune thyroiditis: a cross-sectional study based on two Chinese communities with different iodine intake levels. Eur J Endocrinol (2011) 164(6):943–50. doi: 10.1530/EJE-10-1041 21444648

[25] Liu Y Liu S Mao J Piao S Qin J Peng S. Serum trace elements profile in graves’ disease patients with or without orbitopathy in northeast China. BioMed Res Int (2018) 3029379. doi: 10.1155/2018/3029379 29546054 PMC5818896

[26] Bulow PI Knudsen N Carle A Schomburg L Köhrle J Jørgensen T. Serum selenium is low in newly diagnosed graves’ disease: a population-based study. Clin Endocrinol (Oxf) (2013) 79:584–90. doi: 10.1111/cen.12185 23448365

[27] Aihara K Nishi Y Hatano S Kihara M Yoshimitsu K Takeichi N. Zinc, copper, manganese, and selenium metabolism in thyroid disease. Am J Clin Nutr (1984) 40(1):26–35. doi: 10.1093/ajcn/40.1.26 6741853

[28] Erdal M Sahin M Hasimi A Uckaya G Kutlu M Saglam K. Trace element levels in hashimoto thyroiditis patients with subclinical hypothyroidism. Biol Trace Elem Res (2008) 123(1–3):1–7. doi: 10.1007/s12011-008-8117-8 18322655

[29] Rostami R Nourooz-Zadeh S Mohammadi A Khalkhali HR Ferns G Nourooz-Zadeh J. Serum selenium status and its interrelationship with serum biomarkers of thyroid function and antioxidant defense in hashimoto’s thyroiditis. Antioxidants (Basel) (2020) 9(11):1070. doi: 10.3390/antiox9111070 33142736 PMC7692168

[30] Nourbakhsh M Ahmadpour F Chahardoli B Malekpour-Dehkordi Z Nourbakhsh M Hosseini-Fard SR. Selenium and its relationship with selenoprotein p and glutathione peroxidase in children and adolescents with hashimoto’s thyroiditis and hypothyroidism. J Trace Elem Med Biol (2016) 34:10–4. doi: 10.1016/j.jtemb.2015.10.003 26854239

[31] Federige MAF Romaldini JH Miklos ABPP Koike MK Takei K Portes ES. Serum selenium and selenoprotein-p levels in autoimmune thyroid disease patients in a select center: a transversal study. Arch Endocrinol Metab (2017) 61(6):600–7. doi: 10.1590/2359-3997000000309 PMC10522059 29412385

[32] Mseddi M Gargouri B Mnif F Abid M Guermazi F Attia H. Involvement of the selenium level in plasma glutathione peroxidase activity in newly diagnosed patients with grave’s disease and hashimoto thyroiditis. J Adv Biotechnol (2016) 5(3):761–7. doi: 10.24297/Jbt.V5i3.1505

[33] Kochman J Jakubczyk K Bargiel P Janda-Milczarek K. The influence of oxidative stress on thyroid diseases. Antioxidants (2021) 10(9):1442. doi: 10.3390/antiox10091442 34573074 PMC8465820

[34] Onal H Keskindemirci G Adal E Ersen A Korkmaz O. Effects of selenium supplementation in the early stage of autoimmune thyroiditis in childhood: an open-label pilot study. J Pediatr Endocr Metab (2012) 25:639–44. doi: 10.1515/jpem-2012-0078 23155687

[35] Gabulov GG Jabrailova GI. The effect of selenium on the immune status in the complex treatment of children with autoimmune thyroiditis. Russian Bull Perinatology Pediatr (2019) 64(2):87–93 (In Russ.). doi: 10.21508/1027-4065-2019-64-2-87-93

[36] Fairweather-Tait SJ Bao Y Broadley MR Collings R Ford D Hesketh JE. Selenium in human health and disease: an overview. Antioxid Redox Signal (2011) 14(7):1338–42. doi: 10.1007/978-3-319-95390-8_1 20812787

[37] Beckett GJ Arthur JR. Selenium and endocrine systems. J Endocrinol (2005) 184(3):455–65. doi: 10.1677/joe.1.05971 15749805

[38] Duntas LH. The role of selenium in thyroid autoimmunity and cancer. Thyroid (2006) 16(5):455–60. doi: 10.1089/thy.2006.16.455 16756467

[39] Wang W Mao J Zhao J Lu J Yan L Du J. Decreased thyroid peroxidase antibody titer in response to selenium supplementation in autoimmune thyroiditis and the influence of a selenoprotein p gene polymorphism: a prospective, multicenter study in China. Thyroid (2018) 28(12):1674–81. doi: 10.1089/thy.2017.0230 30398407

[40] Hisbiyah Y Endaryanto A Setyoboedi B Rochmah N Faizi M Wungu CDK. Effectiveness of selenium supplementation in children with autoimmune thyroiditis: a systematic review and meta-analysis. Int J Health Sci (2022) 6(S9):1395–410. doi: 10.53730/ijhs.v6nS9.12765

[41] Hu Y Feng W Chen H Shi H Jiang L Zheng X. Effect of selenium on thyroid autoimmunity and regulatory T cells in patients with hashimoto’s thyroiditis: a prospective randomized-controlled trial. Clin Transl Sci (2021) 14(4):1390–402. doi: 10.1111/cts.12993 PMC8301566 33650299

[42] Mazokopakis EE Chatzipavlidou V. Hashimoto’s thyroiditis and the role of selenium. current concepts. Hell J Nucl Med (2007) 10(1):6–8. 17450242

[43] Nève J. Human selenium supplementation as assessed by changes in blood selenium concentration and gluthathione peroxidase activity. J Trace Elem Med Biol (1995) 9):65–73. doi: 10.1016/S0946-672X(11)80013-1 8825978

[44] Ashton K Hooper L Harvey LJ Hurst R Casgrain A Fairweather-Tait SJ. Methods of assessment of selenium status in humans: a systematic review. Am J Clin Nutr (2009) 89):2025S–39S. doi: 10.3945/ajcn.2009.27230F 19420095

[45] Bermingham EN Hesketh JE Sinclair BR Koolaard JP Roy NC. Selenium-enriched foods are more effective at increasing glutathione peroxidase (GPx) activity compared with selenomethionine: a meta-analysis. Nutrients (2014) 6(10):4002–31. doi: 10.3390/nu6104002 PMC4210904 25268836

[46] Guaraldi F Giaccherino R. Endocrine autoimmunity in down’s syndrome. Front Horm Res (2017) 48:133–46. doi: 10.1159/000452912 28245458

[47] Cutler AT Benezra-Obeiter R Brink SJ. Thyroid function in young children with down syndrome. Am J Dis Child (1986) 140(5):479–83. doi: 10.1001/archpedi.1986.02140190089034 2938470

[48] Ivarsson SA Ericsson UB Gustafsson J Forslund M Vegfors P Annerén G. The impact of thyroid autoimmunity in children and adolescents with down syndrome. Acta Paediatr (1997) 86(10):1065–7. doi: 10.1111/j.1651-2227.1997.tb14808.x 9350885

[49] Graber E Chacko E Regelmann MO Costin G Rapaport R. Down syndrome and thyroid function. Endocrinol Metab Clin N Am (2012) 41:735–45. doi: 10.1016/j.ecl.2012.08.008 23099267

[50] Shalitin S Phillip M. Autoimmune thyroiditis in infants with down’s syndrome. J Pediatr Endocrinol Metab (2002) 15(5):649–52. doi: 10.1515/JPEM.2002.15.5.649 12014525

[51] Johnson MB De Franco E Greeley SAW Letourneau LR Gillespie KM International DS-PNDM Consortium. Trisomy 21 is a cause of permanent neonatal diabetes that is autoimmune but not HLA associated. Diabetes (2019) 68(7):1528–35. doi: 10.2337/db19-0045 PMC6609990 30962220

